# River Basin Cyberinfrastructure in the Big Data Era: An Integrated Observational Data Control System in the Heihe River Basin

**DOI:** 10.3390/s21165429

**Published:** 2021-08-11

**Authors:** Jianwen Guo, Minghu Zhang, Qingsheng Shang, Feng Liu, Adan Wu, Xin Li

**Affiliations:** 1 Key Laboratory of Remote Sensing of Gansu Province, Northwest Institute of Eco-Environment and Resources, Chinese Academy of Sciences, Lanzhou 730000, China; liufeng@lzb.ac.cn (F.L.); wuadan@lzb.ac.cn (A.W.); 2 Jiangsu Center for Collaborative Innovation in Geographical Information Resource Development and Application, Nanjing 210023, China; 3 School of Computer and Communication Technology, Lanzhou University of Technology, Lanzhou 730050, China; zhangmh@lut.cn; 4 School of Information Engineering, Lanzhou University of Finance and Economics, Lanzhou 730020, China; shangqsh@lzufe.edu.cn; 5 National Tibetan Plateau Data Center, Institute of Tibetan Plateau Research, Chinese Academy of Sciences, Beijing 100101, China; xinli@itpcas.ac.cn; 6 CAS Center for Excellence in Tibetan Plateau Earth Sciences, Chinese Academy of Sciences, Beijing 100101, China

**Keywords:** Heihe River, river basin cyberinfrastructure, wireless sensor networks, automated observational data integration

## Abstract

River basin cyberinfrastructure with the Internet of Things (IoT) as the core has brought watershed data science into the big data era, greatly improving data acquisition and sharing efficiency. However, challenges in analyzing, processing, and applying very large quantities of observational data remain. Given the observational needs in watershed research, we studied the construction of river basin cyberinfrastructure and developed an integrated observational data control system (IODCS). The IODCS is an important platform for processing large quantities of observational data, including automated collection, storage, analysis, processing, and release. This paper presents various aspects of the IODCS in detail, including the system’s overall design, function realization, big data analysis methods, and integrated models. We took the middle reaches of the Heihe River Basin (HRB) as the application research area to show the performance of the developed system. Since the system began operation, it has automatically received, analyzed, and stored more than 1.4 billion observational data records, with an average of more than 14 million observational data records processed per month and up to 21,011 active users. The demonstrated results show that the IODCS can effectively leverage the processing capability of massive observational data and provide a new perspective for facilitating ecological and hydrological scientific research on the HRB.

## 1. Introduction

Big data, first proposed by Alvin Toffler in The Third Wave, is characterized by broad sources, large volumes, multiple modes, and high values, and it is changing how people understand the world [1,2,3]. Big data has started to support the rapid development of Earth sciences [4,5,6]. Specifically, it has begun to play an increasingly important role in the research of Earth surface system sciences, which mainly focus on the overall characteristics of the interaction among the elements in the hydrosphere, pedosphere, atmosphere, and biosphere [7,8,9]. Therefore, big data can well support the integrated research of regional ecological economy [10]. After years of the constant construction of Earth surface observational systems, massive quantities of observational data have been accumulated into big Earth data at a considerable scale. In particular, with the rapid development of technologies related to the Internet of Things (IoT) [11,12], observational systems on the Earth’s surface are becoming increasingly mature, with the quantity of observational data reaching the petabyte scale and the number of modes of observational data significantly increasing [11,13,14]. The rise of the IoT has made it possible to acquire Earth surface observational and monitoring data in real time. We reviewed, compared, and analyzed several typical instances of data processing systems for big data, as shown in Table 1. Most of these systems were constructed for data delivery, storage, and visualization. However, unified standards and integrated systems of descriptions, organization, transmissions, interfaces, management, and applications of massive quantities of observational data are missing. This issue has become one of the most significant challenges for managing and sharing big Earth data [15,16,17]. In particular, regarding observational data from the IoT, which are usually termed streaming data and feature high speeds, large volumes, and uncertainties, traditional data reception, management, and visualization are confronted with challenges. Therefore, we must develop fully automated data receiving, intelligent data quality control, and more efficient and convenient data management and visualization methods.

Cyberinfrastructure (CI) is the bridge between information engineering and scientific research [26]. With the help of information technology and network technology, CI integrates observational instruments, storage devices, computing devices, and other resources to provide more efficient, faster, and more flexible collaboration space for scientific research. Therefore, as an application instance of CI—an integrated observational data control system (IODCS) that combines the IoT, wireless sensor networks (WSNs), cloud computing, and data visualization—is developed in this paper. The IODCS is considered a standardized system that makes it possible to achieve the automated reception and storage, distributed storage, quality control, security sharing, and visualized analysis of observational data according to descriptive data specifications. For instance, if the observational data obtained by each observational system could be automatically received and stored according to the database index type and data format, data storage efficiency would be improved. The introduction of distributed storage into the system serves as important technical support for the scientific and efficient management of massive quantities of observational data. Developing and integrating automated data quality control can achieve automated anomaly detection and observational data processing. Moreover, data sharing is an important driving force for promoting Earth surface research, and online data visualization allows users to more intuitively and conveniently analyze and understand data.

In this article, the authors present a new CI application framework for automatic observational networks and demonstrate it in the Heihe River Basin (HRB), a typical endorheic river basin in Northwest China. The new CI application framework facilitates scientific observational and data service related to the river basin and brings new insights into applications in other areas.

## 2. Description of the Proposed System

### 2.1. System Overview

**Figure 1:**
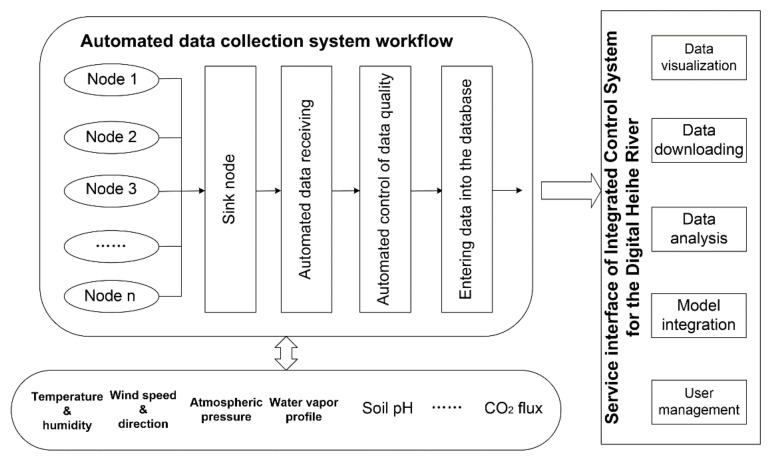
Framework of the IODCS.

The IODCS was designed to provide a universal data sharing platform for basin-scale scientific studies. The system is composed of the following functions: automated data reception and storage, automated data quality control, distributed data storage, data sharing services, model integration, and visualization. Figure 1 shows the framework of the IODCS, and the details are presented as follows.

### 2.2. Automated Data Reception and Storage

The automated data reception module aims to normalize different data organization modes of observational devices from different sources. Through the programming of the server-side interface of the specific WSN observational device with the software development kit (SDK) [27], the data string transmitted to the server is directly intercepted, and the data are then reorganized as a defined standard data string. The normalized data string simplifies the back-end automated data preprocessing and warehousing procedure.

**Figure 2:**
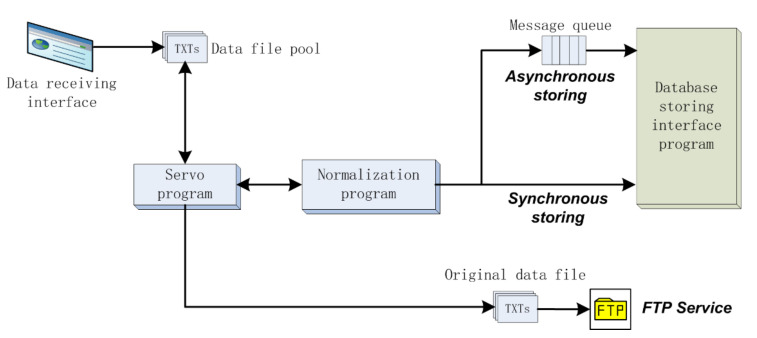
Process of the automatic receiving, normalizing, and storing of observational data in the IODCS.

During the automated data reception process, the data status of the WSN on the nodes is automatically monitored, collected, and normalized in an unattended manner. The observational equipment regularly sends the observational data in the local cache to the data receiving interface program on the remote server. The server can also send instructions to request data uploading from the observational equipment. Figure 2 shows the process.

The servo program automatically polls whether the data files in the data file pool have changed. When there are new data, the data reception process automatically starts, and the obtained data are pushed to a normalization program. After normalization, the disordered multisource data string becomes a simple and standard data string that is then sent to the database storage interface program. The data storage process has no human intervention, and it provide a unified database access interface for various data reception programs.

The automated storage process of observational data can be accomplished synchronously or asynchronously based on the actual needs of the system. In the synchronous mode, upon receiving the data, the normalization program invokes the storage program to immediately store the observational data in the database. In the asynchronous mode, the normalization program puts data into a message queue, and then the storage program obtains data from the message queue and stores them in the database when the system is not busy.

### 2.3. Automated Data Quality Control

**Figure 3:**
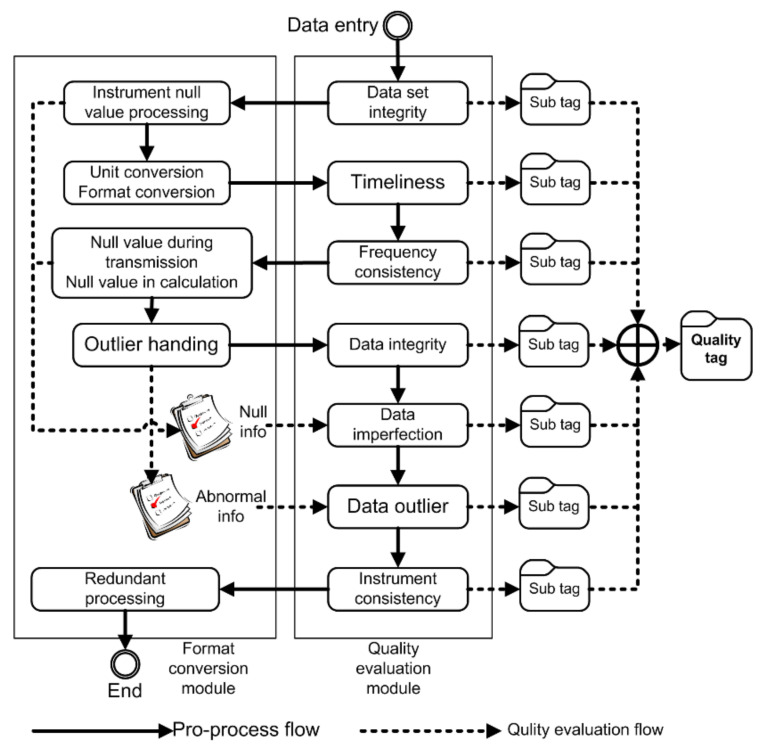
Data quality control items and flow in the IODCS.

For automated observational data, errors, such as source errors, calibration errors, and transmission errors, occur. Source errors are caused by the observational device itself, calibration errors occur during the calibration and correction of observational data, and transmission errors consist of the operational errors and interference errors incurred during data transmission [28,29,30,31,32]. The data quality control model is deployed in the IODCS to evaluate and control the quality of the observational data using unified standards and a consistent quality control system during the generation, processing, and transmission of data. Moreover, the transformation model deployed in the IODCS describes the conversion type factors during automated data processing, which contributes to the application of a consistent conversion system in the automated process. Additionally, automated observational devices arranged in the field are produced by different manufacturers and use different parameters and data storage formats. Thus, these differences should be converted before data storage to guarantee that the data are stored with unified standards, namely semantics and format. The automated conversion algorithm and the quality evaluation algorithm are sequentially executed in the logical flow of data processing [33]. Figure 3 shows a flow chart of automated data quality control.

There are two kinds of control logic processes in the data quality control flow: format conversion and quality evaluation. The format conversion process mainly deals with possible format problems in observational data. Quality evaluation evaluates the quality status of data and attaches the results as a label. These two processes include a variety of different methods or algorithms to process observational data. The details are shown in Table 2.

### 2.4. Distributed Storage System

**Figure 4:**
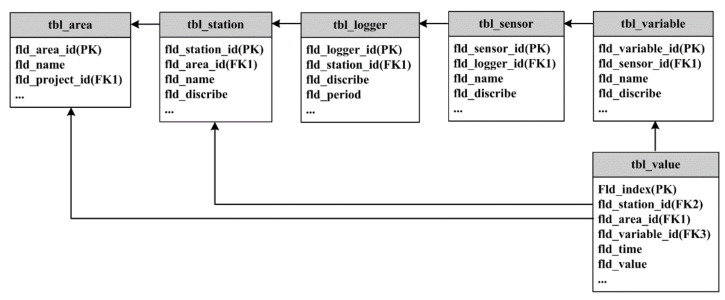
Conceptual structure of the relational observational database in the IODCS.

In the past, observational data were mainly stored in the form of text or spreadsheets. Since the data files from different sources contained different contents and were in different formats, the efficiency of data management, analysis, and use was rather low. As the automated observational data stored and managed in an observational database are strongly structured, the relational database was selected as the basis of storage design in this study, and the Greenplum distributed database [34,35] was used for data storage. After fully investigating and analyzing the observational projects and observational elements that had been carried out or were planned, we designed and constructed the IODCS with an object-oriented relational database design method. The new data storage and management mode significantly improved efficiency. Figure 4 shows the structure of the relational database designed in this study.

**Figure 5:**
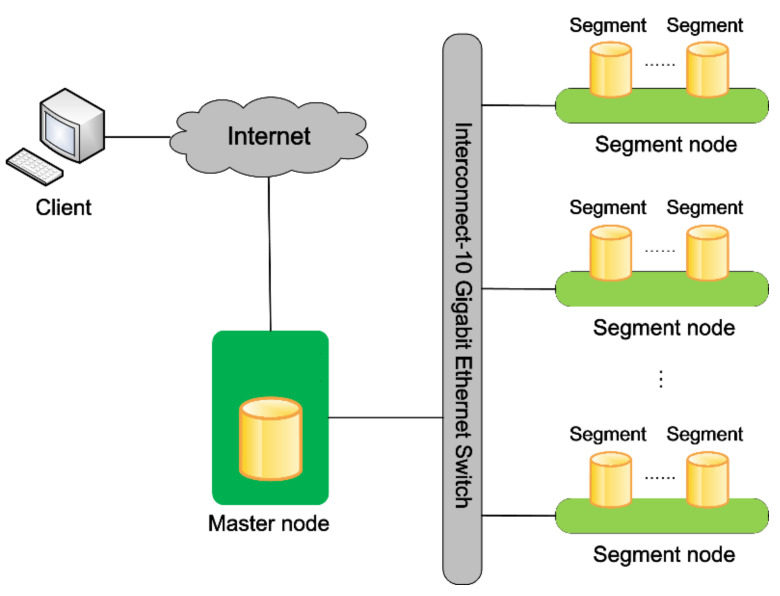
A high-performance computer cluster for the distributed observational database.

As shown in Figure 5, we built a high-performance computer cluster with one master node, multiple segment nodes, and a Greenplum distributed observational database. The number of segment nodes can be dynamically expanded according to actual needs. The master node is responsible for organizing and dispatching the cluster operation and connecting with the external network. All data are stored on segment nodes. Each segment node can contain multiple segments. A segment is the basic unit for performing database-concurrent operations. The number of segments per segment node may be different according to user requirements and server hardware performance. This kind of cluster application mode has excellent advantages in data query efficiency, especially when the quantity of query data is large [36].

### 2.5. Model Integration

**Figure 6:**
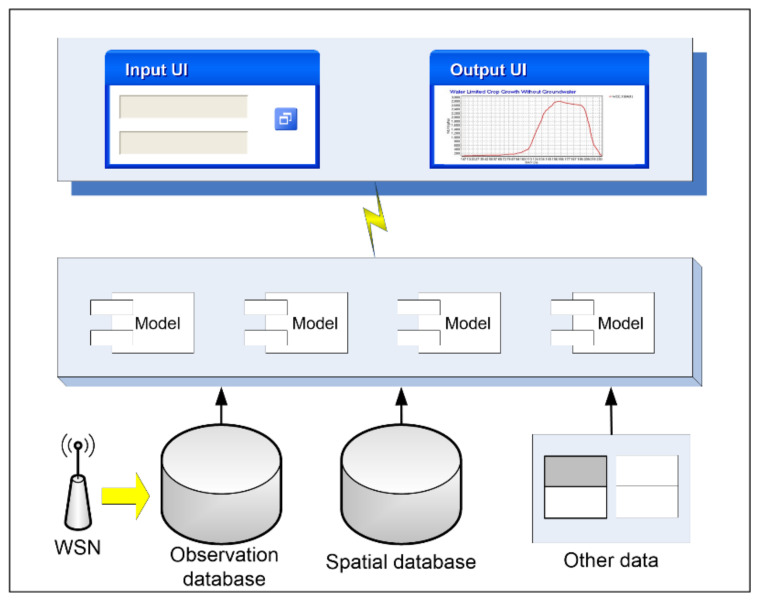
The logic architecture of model integration in the IODCS.

An essential function of IODCS is to integrate the online scientific model. Establishing a scientific model for the HRB and its surrounding areas is an indispensable approach to basin-scale scientific research. Researchers have developed different ecological and hydrological models for the Heihe River Basin. To efficiently combine these models with real-time observational data and to more quickly serve basin research, we developed an online model integration module in the IODCS. The models are integrated with the model integration module through a web service interface. As shown in Figure 6, the relevant models are called by the IODCS through the online integration module, and the input parameters, output results, real-time observational data, and basic spatial data support of the model are controlled by the IODCS.

By inputting the needed parameters of the integrated model via the visual interface of the IODCS, users can obtain the real-time predicted results online.

### 2.6. Visualizations

The IODCS has many functions for observational data, including automated reception and storage, automated format conversion and quality evaluation, statistical analysis, and classification according to standard data description and relevant rules. All these functions are intended to provide convenience for users, and every bit of the system’s information is vital. Thus, we developed a visualization module to intuitively show this information.

**Figure 7:**
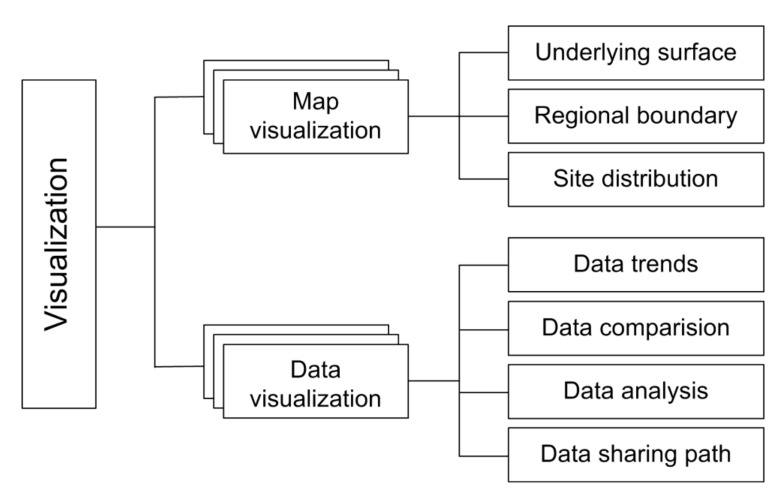
Data visualization function system in the IODCS.

The data visualization module was developed using the OpenLayers plug-in and Apache ECharts control [37,38,39]. Figure 7 shows the functional structure of the visualization system. The map visualization function provides visualization support for the underlying surface, regional boundary, and distribution of the observational stations. The data visualization function provides visualization support for data trends, data comparison, data analysis, and data sharing paths. With the help of the visualization module, we can easily check the location and configuration of the observational stations and the type and data trends of the observational variables online. Moreover, the observational data can be intuitively analyzed online in real time.

The IODCS optimizes the technical process of data visualization to avoid the performance loss caused by excessively frequent connection and visualization requests between the client and server. Based on WebSocket [40], the IODCS establishes a continuous long-term connection between the client browser and the server, which not only reduces the server load but also constantly provides the client with the latest data and their trends through data visualization. In other words, once the server receives the new field data, the client immediately updates the data.

## 3. Application in the Heihe River Basin

### 3.1. Case Study Area and the Overall Implementation

**Figure 8:**
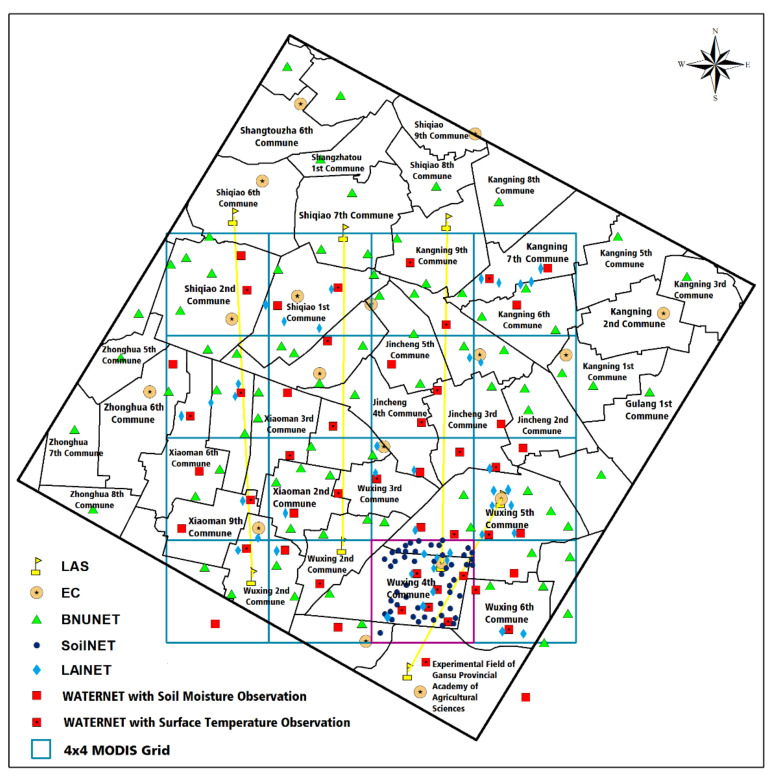
Layout of the WSN observational nodes in the middle reaches of the HRB [43].

The Heihe Watershed Allied Telemetry Experimental Research (HiWATER) is a large-scale comprehensive observational experiment [41,42,43,44] in which midstream observations were launched in 2012. To effectively capture the spatial heterogeneity of the surface elements in the river basin and to verify the authenticity of remote sensing data, HiWATER was carried out in a 5.5 × 5.5 km core observational area located in the Yingke/Daman Irrigation District in the middle reaches of the Heihe River. The spatial optimization algorithm selected a total of 198 observational stations. With the WSN as the bridge, a great variety of meteorological, hydrological, and ecological observational projects with multisource sensors densely distributed on the scale of the river basin/irrigation district were integrated to establish a fully automated observational system with spatiotemporally coordinated ecohydrological sensors. Figure 8 shows the layout of the WSN observational nodes in the middle reaches of the HRB.

The core observational area was equipped with four types of surface variable observational devices with remote wireless data transmission, namely SoilNET for observing the soil moisture/temperature, WATERNET for observing the soil moisture/temperature and surface temperature, LAINET for observing the leaf area index, and AWS (automatic weather stations), as shown in Table 3. The nodes of BNUNET (designed by Beijing Normal University for observing the soil moisture/temperature), LAS (large aperture scintillometer), and EC (eddy covariance system) have no remote wireless data transmission functionality.

The IODCS automatically collected more than 300 observational variables (observational variables at different heights or depths were regarded as different observational variables), with a 10 min sampling period for each type of observational variable. During the observational period of the synergetic enhancement between the Earth and the satellite, the sampling period was 1 min, and a small number of other observational variables were sampled every 30 min [45,46]. In this study, the designed fully automated observations and IODCS comprehensively improved the overall observational ability, information level, and observational data sharing service for the ecohydrological processes in the river basin.

We have demonstrated that our system developed for HiWATER and its online observational data support platform can provide observational managers and researchers with online data services, including the visualization of two-dimensional, three-dimensional, or multidimensional geoscience data; on-demand data downloading; the automatic generation of observational inspection reports and FTP support; and computing services, such as data-aided analysis, geographic information system (GIS) spatial support, and professional model analysis.

### 3.2. Data Management and Service

**Figure 9:**
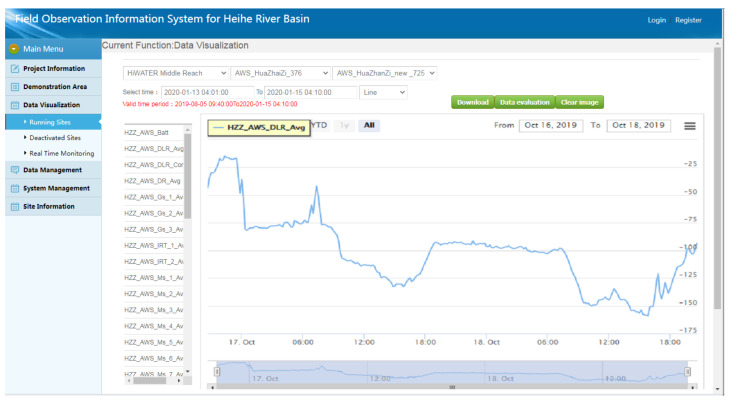
Data management and service interface of the IODCS of the HRB (translated from the user interface in Chinese).

Figure 9 shows the data service interface for data management and release. To date, the IODCS of the HRB has automatically received, processed, and stored more than 1.4 billion observational data records, with an average of over 14 million observational data records per month. In August 2012, the month in which the most intensive observations were carried out, the number of stored observational data records reached 340 million. On the premise of efficiently completing the automated preprocessing and distributed storage of observational data, the IODCS provides observers and researchers with flexible and convenient practical functions such as online data visualization, online data querying, on-demand data downloading, alarms for anomalies in observational devices, and the automated generation of daily equipment inspection reports. The function of the daily equipment inspection reports is to automatically sort the observational data generated by all the observational equipment in the HRB observational network and generate reports every day, which can help users intuitively understand the operational status of the observational network and the quality of the observational data.

### 3.3. Real-Time Online Data Browsing and Analysis

**Figure 10:**
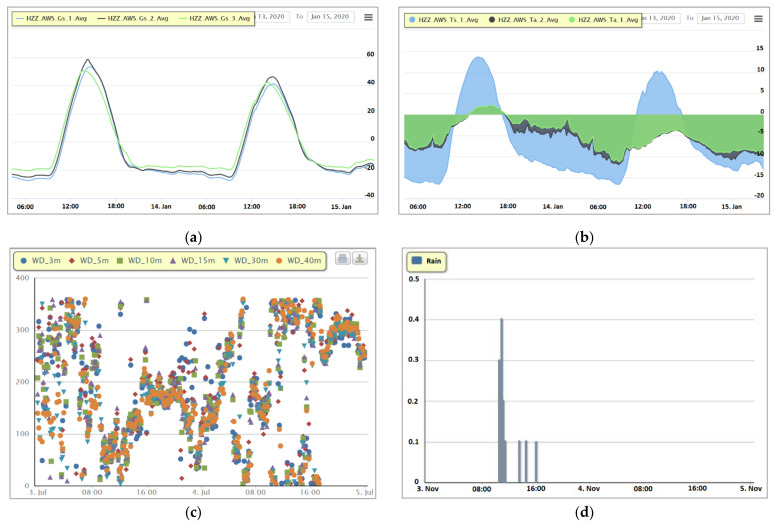
Different online visualization forms of the observational data in the HRB. (**a**) A curve chart example of soil heat flux (in this example, in a corn field near Daman station); (**b**) an area chart example of soil temperature (in this example, 2, 4, and 6 cm underground near Daman station); (**c**) a scatter chart example of wind direction (in this example, near Dashalong station on 4 November 2019); (**d**) a column chart example of rainfall (in this example, near Dashalong station on 4 November 2019).

As shown in Figure 10, during the observational period in the HRB, the observational data are collected every 10 min (1 min in intensive mode), and the related data curves are also automatically updated every 10 min (1 min in intensive mode). Users can arbitrarily call and display the data of any observational elements at any observational station during any observational period and browse the data in various online visualization formats, e.g., curve charts, area charts, scatter charts, or column charts. Moreover, the system supports the multivariable comparative analysis of observational data. Users can combine multiple correlating variables together for the browsing of visual contrast.

### 3.4. Data Downloading on Demand

**Figure 11:**
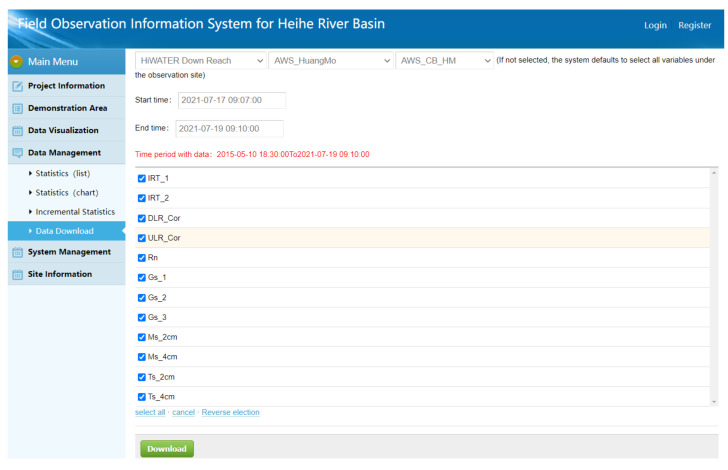
Data downloading interface (translated from the user interface in Chinese).

The application of relational databases facilitates the on-demand downloading of observational data. In the past, observational data in the HRB were shared through data files. Each file stored some observational elements obtained in specific areas within a given period in a certain format. After gaining the shared data files, researchers extract and sort the data that they need from many data files based on their research demands. This approach is very inefficient and time consuming. To solve this problem, the authors of this study developed the IODCS in the HRB so that researchers may directly obtain the observational elements collected from a specific area within the required time interval from the system according to their research needs while keeping data acquisition process simple and efficient. Figure 11 shows the data downloading interface.

### 3.5. Intelligent Analysis of the Status of the Observational Network

**Figure 12:**
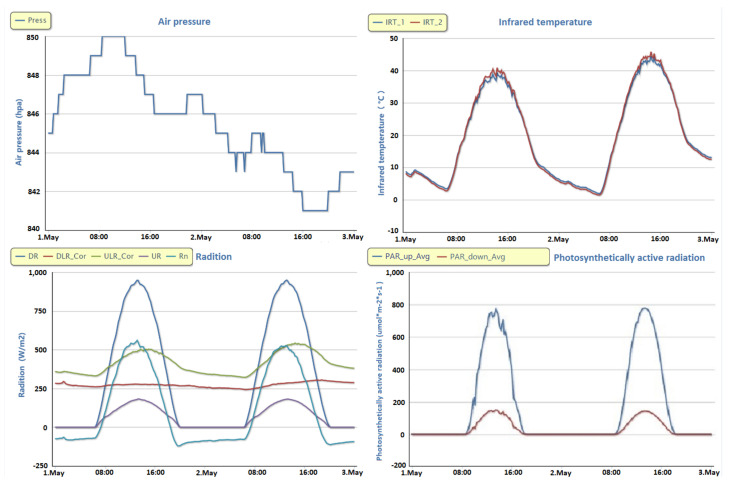
Part of the equipment status inspection report of the IODCS.

Because there are many observational nodes and elements in the observational area, it is very important to know the status of the observational network in real time. The IODCS can automatically collect and organize the observational data of all nodes in the whole observational network every day and generate a graphical status report of the observational network. This report can help maintenance personnel monitor the operational status of the observational system and analyze whether there are errors in the data. Figure 12 shows part of the equipment status inspection reports.

### 3.6. Anomaly Detection

The IODCS applies some data analysis methods to assist in the analysis and detection of observational data. For example, a fitting method is used to detect abrupt anomalies in the data. An artificial neural network analyzes the abnormal fluctuation of the data, and the integrity of the dataset is analyzed using a statistical method.

**Figure 13:**
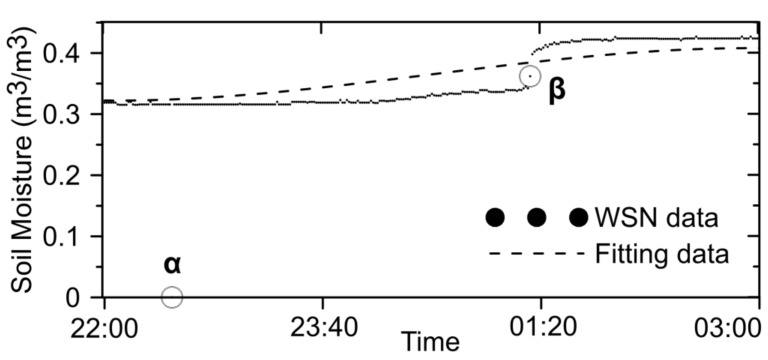
Detection of data exceptions using the fitting method.

Figure 13 shows the detection of data exceptions using the polynomial curve fitting method (using the same strategy as MATLAB; see https://www.mathworks.com/help/matlab/ref/polyfit.html (accessed on 28 June 2021)). Considering the WSN data for soil moisture within a certain period as an example [47], according to the time-series scatter diagram and the fitted results of the sample data, the observed soil moisture value sharply increased at approximately 01:20:00 on 26 June 2012. On that day, due to the change (rain) in the external environment, the fitted data revealed the fluctuation well.

The data at point β might be misjudged as abnormal data if there were accidental errors but can be correctly identified among the quality control elements with abnormal fluctuation. However, with a residual error of 0.3135, which was much greater than three standard deviations (standard deviation = 0.0278), the data at point α were abnormal.

**Figure 14:**
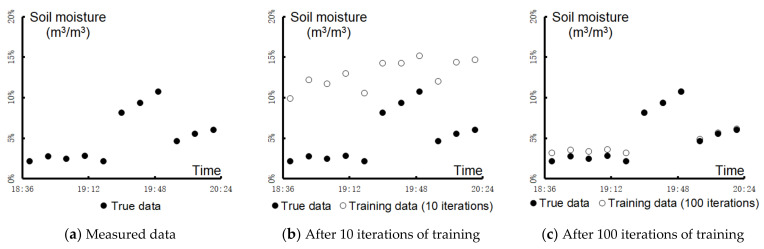
Analysis of data exceptions using the BP neural network method.

Figure 14 shows the analysis of data exceptions using the backpropagation (BP) neural network method. Taking the measured WSN data for soil moisture on a specific day as an example, we analyzed the fluctuation in the observational data. Figure 14 illustrates that as the number of training iterations increased, the model’s accuracy was greatly improved. Then, we used the true data range from 18:36 to 20:24 as the samples by which the BP algorithm was applied to train the network, and the corresponding weight matrixes and their eigenvectors were calculated. By comparing these eigenvectors with the standard eigenvector, e.g., the eigenvector of the data collected during a rain spell, researchers could determine whether data are abnormal.

### 3.7. Online Computing with Integrated Models

We used online calculations for growth monitoring and yield prediction [48,49] and soil moisture spatial interpolation of crops as examples of these integrated models [50,51]. Table 4 shows the details of the integrated models.

**Figure 15:**
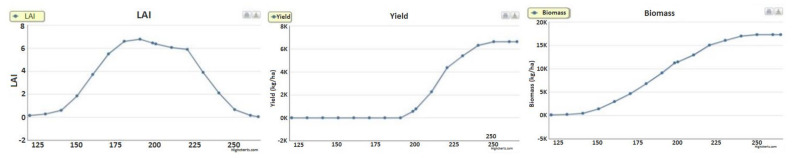
Prediction results of the crop growth model.

**Figure 16:**
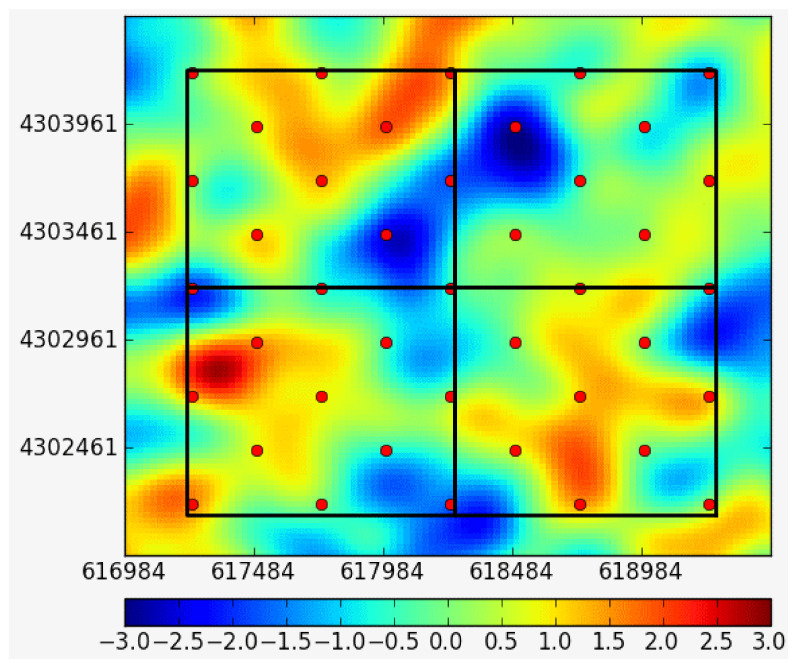
Online calculation results of the soil moisture spatial interpolation model (using discrete soil moisture observational data as the input, the platform calls the online spatial interpolation model to calculate the spatial distribution data for soil moisture).

The predicted results of crop growth, yield, and biomass are shown in Figure 15. The predicted results of the soil moisture spatial interpolation model are shown in Figure 16.

## 4. Summary and Outlook

The IODCS developed in this paper is a highly standardized, strongly interactive, secure, and reliable instance of CI application. The HRB area application shows that the IODCS has completely changed the method of storing and managing observational data using data files by applying a relational database to manage strongly structured observational data, which greatly increases data management efficiency. The change in the data management mode also directly influences the subsequent use of observational data, not only making it possible to efficiently query, analyze, and download the observational data but also remarkably improving the method and efficiency of data service and sharing. The IODCS facilitates other application modes for observational data, such as online model integration application and the intelligent analysis of the status of the observational network.

However, due to the current level of automated data processing, only a limited number of automated preprocessing algorithms are used in the IODCS, which, to a certain degree, confines the processing capacity of the CI system. To meet the demands of scientific field observations in the big data era, the IODCS must be further optimized to combine big data with machine learning and deep learning to provide a one-stop platform for data management and model development for massive observational data to support big Earth data sharing services for scientific research in the HRB.

## Figures and Tables

**Table 1:** Typical instances of big data processing systems.

No.	System Name	Year	Main Functions	References
1	BigDansing	2015	A big data cleaning system to tackle efficiency, scalability, and ease-of-use issues in data cleaning; it can be run on most general-purpose data processing platforms, ranging from DBMSs to MapReduce-like frameworks.	[18]
2	SWITCH	2019	It offers a flexible co-programming architecture that provides an abstraction layer and an underlying infrastructure environment, which can help to both specify and support the life cycle of time-critical cloud native applications.	[19]
3	DRIP	2019	It was developed for the dynamic optimization of data services in research support environments and might be used for a number of similar applications involving distributed services and large, dynamic datasets with further investigation and development.	[20]
4	SPS-IUTO	2020	To achieve significant improvements in terms of energy and redundant data, a matrix completion-based sampling point selection joint intelligent unmanned trajectory optimization (SPS-IUTO) scheme for unmanned aerial vehicles (UAVs) was proposed to plan sampling points for UAVs in both time and space.	[21]
5	BD-VTE	2020	A novel baseline data based verifiable trust evaluation (BD-VTE) scheme was proposed to guarantee security at a low cost for massive data. The BD-VTE scheme includes a verifiable trust evaluation (VTE) mechanism, an effectiveness-based incentive (EI) mechanism, and a secondary path planning (SPP) strategy, which are used for reliable trust evaluation, reasonable reward, and efficient path adjustment, respectively.	[22]
6	DRMCS	2020	DRMCS, a data collection scheme for mobile crowdsensing vehicular networks, was proposed to enhance the data collection rate in vehicular networks for opportunistic communication.	[23]
7	SDAC	2021	A novel secure and dynamic access control (SDAC) model was developed for IoT networks (smart traffic control and roadside parking management). It allows IoT devices to securely communicate and share information through busing wired and wireless networks (cellular networks or Wi-Fi).	[24]
8	aiRe	2021	This open-access tool simplifies air quality data analysis and visualization, with the desirable effects of removing ownership costs, fostering appropriation by nonexpert users, and ultimately promoting informed decision making for the general public and local government authorities.	[25]

**Table 2:** Format conversion and quality evaluation methods used in the IODCS system.

Method	Type	Function	Impact on Data
Instrument null value	Format conversion	Detect null values caused by the instrument	Depending on the strategy, the data may be modified
Unit and format conversion	Format conversion	Detect and handle unit and format inconsistencies in observational data	The data will be modified
Null value during transmission and calculation	Format conversion	Detect and handle null values caused by other reasons	Depending on the strategy, the data may be modified
Outlier	Format conversion	Detect and handle data that do not adhere to data trends	Depending on the strategy, the data may be modified
Redundant processing	Format conversion	Detect and delete duplicate data based on timestamps	The duplicate data will be deleted
Dataset integrity	Quality evaluation	Check whether all the variables of integrated observations have observational values. For example, is there a missing value in a temperature profile?	The data will not be modified but will be tagged
Timeliness	Quality evaluation	Check the timeliness of warehousing data	No data will be modified, but the system will tag the variable
Frequency consistency	Quality evaluation	Check whether the data are collected according to the acquisition frequency	No data will be modified, but the system will tag the variable
Data integrity	Quality evaluation	Tag the data according to the result of outlier detection	No data will be modified, but the system will tag the variable
Data imperfection	Quality evaluation	Tag the data according to the result of null value detection	No data will be modified, but the system will tag the variable
Data outlier	Quality evaluation	Tag the abnormal data according to the result of outlier detection	No data will be modified, but the system will tag the variable
Instrument consistency	Quality evaluation	Detect abnormal data caused by instrument	No data will be modified, but the system will tag the variable

**Table 3:** Observational devices installed in the core observational area, their related observational variables, and their wireless communication modes.

Device Type	Number of Nodes	Main Observational Variables	Communication Mode
SoilNET	50	soil moisture/temperature	ZigBee, GPRS/3G/4G
WATERNET	55	soil moisture/temperature/salinity, rainfall, snow depth, air moisture/temperature, and wind speed/direction	GPRS/3G/4G/Radio
LAINET	50	leaf area index	GPRS/3G/4G/Radio
AWS	18	soil moisture/temperature/heat flux, surface temperature air moisture/temperature/pressure, wind speed/direction, and radiation	GPRS/3G/4G/Radio

**Table 4:** Models integrated into the IODCS.

**No.**	**Model**	**Function**	**Development Language**
1	WOFOST crop growth model	With the physiological and ecological processes of crops, e.g., assimilation, respiration, transpiration, and dry matter partitioning, as the simulation basis, the WOFOST crop growth model simulates the growth of crops under the circumstances of potential growth, restricted water, and limited nutrients.	Fortran [52]
2	Spatial kriging interpolation model	After examining soil moisture in the typical irrigated farmland in the upper reaches of the Heihe River as the object of study, relevant extension packs of the Python language are applied to analyze the spatial variability in the observational data and build the spatial kriging interpolation model to estimate the soil moisture in the study area.	Python

## Data Availability

Data available in a publicly accessible repository that does not issue DOIs. Publicly available datasets were analyzed in this study. This data can be found here: [http://210.77.68.221:10066/iframe].
